# Supplementary material for: Bridging disparity in knowledge and utilization of contraceptive methods among married couples in the pastoralist community of Fentale District, Eastern Ethiopia
Source: PLoS One. 2024 Nov 11;19(11):e0309703. doi: 10.1371/journal.pone.0309703 (PMC11554224; doi:10.1371/journal.pone.0309703)
Supplement: Attachment — Submitted filename: Response to Reviewers.docx [file pone.0309703.s001.docx]

**Title: Bridging Disparity in Knowledge and Utilization of Contraceptive Methods among Married Couples in the Pastoralist Community of Fentale District, Eastern Ethiopia**

**Manuscript Number: PONE-D-24-21162**

**Authors:**

Sena Adugna Beyene([senaada491@gmail.com](mailto:senaada491@gmail.com))

Sileshi Garoma([teferabelachew2@gmail.com](mailto:teferabelachew2@gmail.com))

Tefera Belachew([garomaabe@gmail.com](mailto:garomaabe@gmail.com))

Dear Academic Editor and Reviewers,

Thank you for your valuable comments and suggestions, which have greatly improved our study. We have carefully addressed all the points raised and have incorporated the necessary revisions into the manuscript as outlined below.

Editor:

Comment: Reviewers' comments:

Response: Thank you for handling and assigning the manuscript to a knowledgeable reviewer from the same domain. In response to your comments, we have addressed all the queries and clarifications rose by the reviewer.

**Authors Responses for the Reviews Comments July 11, 2024**

| Reviewer#1: Review Feedback | **Authors’ responses** |
| --- | --- |
| 1. : General Comments Grammatical and formatting issues. For example, whether numbering of the section headings adheres to the journal guideline should be checked. There are concerns with the authors’ adherence to the writing conventions mainly characterized by lack of logical flow of ideas within a given paragraph, e.g., paragraph 1 of Introduction, and between paragraphs as well. In another example, the last paragraph of the Introduction is nearly 1 page long and composed of 24 sentences which is far beyond the recommended writing standards. The inclusion and exclusion criteria lack clarity and specificity. | Thank you for your thorough review of our manuscript. We have made the necessary revisions to address grammatical and formatting issues, including section headings and the logical flow of ideas within and between paragraphs. The first paragraph of the Introduction and the last, lengthy paragraph have been revised for clarity and coherence. We have also clarified and specified the inclusion and exclusion criteria. We appreciate your feedback and believe these changes have strengthened our manuscript. If you have further suggestions, please let us know. |
| 2. Title At least, from the results reporting in the abstract section, reflecting the notion of “bridging disparities …” on the title seems to lead readers to a “wrong” expectation, as it doesn’t align with the content or key findings of this study. In other words, with “bridging disparities …” a reasonable expectation would be an interventional study or an implementation type of research. | Thank you for your valuable suggestion. We have incorporated the changes as recommended in the revised manuscript, particularly in the results reported in the abstract section, reflecting the notion of “bridging disparities.” These findings highlight significant disparities in knowledge and utilization of contraceptive methods among married couples in the pastoralist community, emphasizing the need for targeted interventions to bridge these gaps and improve family planning awareness and usage. We have also modified the title of our manuscript to "Bridging Disparity in Knowledge and Utilization of Contraceptive Methods among Married Couples in the Pastoralist Community of Fentale District, Eastern Ethiopia" to better reflect this notion. |
| 3. Abstract  Background: The authors need to clarify the last phrase, i.e., how “adoption among couples” can lead to limited contraceptive coverage, at least, in the Introduction Section of the manuscript (if not in the abstract). | Thank you for your comment.  We have clarified the last phrase in the Introduction Section of the manuscript |
| 4. Abstract Methods: I suggest the phrase “multistage sampling” to be specified or qualified, e.g., whether it was a multistage cluster sampling, or any other type. | Thank you! This study utilized "multistage sampling," which involves multiple stages of sampling techniques. Specifically, our study employed a multi-stage sampling strategy, selecting districts (woredas) as primary sampling units (PSUs) and sub-districts (kebeles) as secondary sampling units (SSUs). A total of 1496 couples were systematically selected. Figure 1 provides a detailed illustration of the multi-stage sampling strategy, including the rationale behind it. I did not find it necessary to explain all this in the abstract methods. Please refer to Figure 1 for more details. |
| 5.Abstract Methods: Check for grammatical errors, e.g., the below statement should be re-written: “Data entered into EPI Data underwent analysis with SPSS (v23.0) and STATA (v14.0), employing descriptive statistics, bivariate analysis, and binary logistic regression to identify predictors of contraceptive knowledge and utilization.” | Thank you for your remark. Regarding data analysis, the statement has been revised as follows: "Data entered into EPI Data underwent analysis with SPSS (v23.0) and STATA (v14.0), employing descriptive statistics, bivariate analysis, and binary logistic regression to identify predictors of contraceptive knowledge." This information has been incorporated into the Abstract under the Methods section. |
| 6.Abstract Methods:  The authors should justify the use of both SPSS and STATA, at least, in the body of the manuscript. | Thank you! Here’s a detailed response that outlines the specific advantages of using both SPSS and STATA, examples of analyses suited to each software, and how their functionalities complement each other in this research. This information has been incorporated into the body of the manuscript under section 2.7, titled "Data Management and Analysis Approach," specifically in paragraphs 2 and 3. |
| 7. Abstract  Results: The first statement is inappropriate for the section, i.e., it is a repetition of the methods, and has nothing to do with results. | Thank you for the suggestion. Your input is essential. We have corrected the duplication by deleting it from the results section in the abstract. Additionally, we have revised the structure of the abstract's results section to ensure clarity and conciseness. |
| 8.Abstract  Results: I don’t think the result “The population, predominantly Oromo (99.6%) and Muslim (97.9%), exhibits educational disparities (53.8% with no formal education).” Is a key finding to appear in the abstract, given the aim of the study. | Of course, I believe this is not a key finding. Especially since our title includes the phrase "Contraceptive Methods," it should specifically highlight the "Disparity in Knowledge and Utilization of Contraceptive Methods" as a key finding in our abstract. Therefore, our abstract's results section has been corrected accordingly. Thank you for your suggestion. I believe that education among couples is a crucial independent variable in our findings, serving as a strong indicator or foundation for significant insights. It would be best to ensure this aspect is thoroughly highlighted. |
| 9. The result “A nomadic pastoralist lifestyle is prevalent among 64.6% of the population.” is also obvious from the title, and seems less important here. In addition, make sure that the classification of nomadic vs other variants of pastoralism is clarified in the main document, given the high frequency that the concept is used in the document, starting from the very first statement of the Introduction Section in the body of the document. | I believe the sentence "A nomadic pastoralist lifestyle is prevalent among 64.6% of the population" is crucial because it specifies the proportion of the pastoralist community in Fentale District that follows a nomadic lifestyle. Our study defines pastoralism as including both nomadic pastoralists (64.6%) and agro-pastoralists (35.4%). The distinction between pastoralism and agro-pastoralism is already described in section 2.7, "Measures." Pastoralism in Ethiopia is well-detailed, so it is important to consider this distinction closely. However, we have omitted the results in the abstract stating “A nomadic pastoralist lifestyle is prevalent among 64.6% of the population” due to the word limit constraints in the abstract. |
| 10. Abstract  Results: I suggest supporting some of the results with statistical analysis outputs. For example, from the result “Women (41.2%) surpass men in contraceptive utilization.” One cannot learn whether the difference between the sex groups was significant or not. | We appreciate your feedback. Identifying the disparity between men and women is a main objective of our research. Therefore, we have revised the abstract results to state "Overall, 27.4% of couples used contraception, with a significant gender difference: 41.2% among women and 13.5% among men." This revision highlights the significant difference in contraceptive utilization between the sexes. |
| 11.Abstract  Results: The contrasting results should be reconciled or explained. For example, 93% of couples didn’t engage in FP discussions, while HEWs played a significant role as information sources and 44.4% of couples demonstrate comprehensive FP knowledge. | To reconcile the contrasting results regarding family planning (FP) discussions, the role of Health Extension Workers (HEWs), and the level of comprehensive FP knowledge among couples, we offer an integrated explanation. Despite 93% of couples not engaging in formal FP discussions, this reflects broader cultural or societal norms where such discussions may be limited. HEWs play a crucial role as information sources, significantly contributing to FP awareness and knowledge in rural and pastoralist communities with limited formal healthcare. While 44.4% of couples demonstrate comprehensive FP knowledge, this does not necessarily correlate with formal FP discussions, as couples may acquire knowledge through HEWs, community health programs, or informal networks. This explanation reconciles the seemingly contrasting findings and provides a comprehensive understanding of FP dynamics within the pastoralist community of Fentale District. Due to word limit constraints, these details are presented in Section 3, "Results," addressing "Socio-demographic and Reproductive Disparities" in the manuscript. |
| 12. Abstract  Results: Check whether “distance from healthcare” or “distance from the nearest health facility” is appropriate. | Both "distance from healthcare" and "distance from the nearest health facility" are generally appropriate, depending on the specific context and clarity desired in the our research. Our study focuses on access to healthcare in a broader sense. "Distance from Healthcare" encompasses various types of healthcare services or facilities, such as clinics, hospitals, or health posts, indicating the distance from any point where healthcare services are available. |
| 13. I suggest revisiting the positive correlations between education, bank account ownership, occupation, and distance from healthcare, and media exposure with knowledge of contraception for the risk of multicollinearity of the independent variables. For example, education, bank account ownership and occupation are very likely to be correlated one other. | Addressing multicollinearity among education, bank account ownership, occupation, distance from healthcare, treatment preferences, and media exposure is crucial in our analysis of contraceptive knowledge. We have employed methods such as assessing Variance Inflation Factors (VIF) to evaluate the degree of correlation between these variables. Our findings indicate that while there is some correlation among these factors, it does not exceed critical thresholds that would invalidate the regression analysis. Nonetheless, we have taken precautions to ensure the robustness of our results by interpreting coefficients cautiously and focusing on their collective influence on contraceptive knowledge. This approach is detailed already under section 2.6, Data Management and Analysis Approach, of the manuscript. |
| 14. How a dependent variable can simultaneously be an independent variable is unclear. Given the title which specifies couples “in the Pastoralist Community”, how nomadic-pastoralist lifestyle was tested is not clear. Unless the phrase is removed from the title, it doesn’t sound logical. | The title of our study, "Bridging Disparity in Knowledge and Utilization of Contraceptive Methods among Married Couples in the Pastoralist Community of Fentale District, Eastern Ethiopia," reflects our focus on understanding and addressing disparities within this specific community. However, we acknowledge the concern about the potential confusion regarding the role of the nomadic-pastoralist lifestyle as a variable in our analysis. To clarify, the nomadic-pastoralist lifestyle is considered an independent variable in our study, influencing knowledge and utilization of contraceptive methods. We analyzed its impact on these outcomes, rather than testing it as a dependent variable. This lifestyle significantly shapes the context within which contraceptive knowledge and utilization occur, providing essential insights into the unique challenges faced by the pastoralist community. We will ensure this distinction is clearly articulated in the manuscript to avoid any confusion. Additionally, we can consider revising the title to "Bridging Disparity in Knowledge and Utilization of Contraceptive Methods among Married Couples in the Fentale District, Eastern Ethiopia" for greater clarity. This modification will focus on the geographical and demographic context without implying the lifestyle as a variable in the title. |
| 15. As indicated in the general comment, none of the results reflects about the essence of “bridging the disparity”. | Thank you for your observation. The essence of "bridging the disparity" in the title is intended to reflect our aim to identify and understand the differences in knowledge and utilization of contraceptive methods among different groups within the pastoralist community, particularly between genders, and to propose potential interventions to address these gaps.  While our results do highlight significant educational disparities and differences in contraceptive knowledge and usage between men and women, we recognize that the title might imply a focus on intervention or policy recommendations to bridge these disparities, which are not explicitly detailed in the results section.  Additionally, we will emphasize in the discussion section how the identified disparities suggest areas for targeted interventions to bridge these gaps, thus maintaining the spirit of the original title while ensuring clarity and accuracy in representing the study's findings. The supplemental information about the disparity among couples is also explained under section 3, "Results," of the manuscript. Overall, this means that this finding is baseline, and the identified disparities among couples suggest areas for targeted interventions to bridge these gaps for the next interventional/educational study. |
| 16.Abstract  Conclusion: Gap in uptake cannot be a reason for low utilization as indicated in the statement, “The limited knowledge and utilization of family planning in the Fentale District may stem from gaps in comprehension, uptake, and disparities among couples.” In other words, uptake is synonymous with utilization. | Thank you! The statement, “The limited knowledge and utilization of family planning in the Fentale District may stem from gaps in comprehension, uptake, and disparities among couples,” implies that gaps in uptake are a separate reason for low utilization. However, since uptake and utilization essentially refer to the same concept, this phrasing is redundant. The key issues contributing to low utilization should be described more accurately. Thus, we have revised the conclusion of the abstract to state: "The limited knowledge and utilization of family planning in the Fentale District stems from gaps in understanding and disparities among couples." This revision is reflected under the conclusion of the abstract of the manuscript. |
| 17. Results: Some of the conclusions are not supported by the results. For example, reproductive history as a factor doesn’t exist in the results. Factors influencing this situation include socio-demographic and reproductive history considerations, with notable variations based on education, occupation, media exposure, bank account ownership, treatment preferences, and healthcare distance. | Thank you! We have gone through the document and made corrections by removing what should be left out and adding what should be added. Please see the revised conclusion under the abstract. |
| 18.Keywords: I suggest revisiting the list for their appropriateness for the content, e.g., dual marginalization, cultural interventions, … | Thank you for the comment. We have revisited the list of keywords to ensure their appropriateness for the content. Please see the revised keywords under the abstract. |
| 18.Introduction Refer to the general comment about numbering of section headings. | **Response:** We appreciate the comment and have ensured consistency. By following this structure, the introduction effectively transitions from the global to the local context, ensuring a logical flow of ideas and adhering to the 'inverted funnel' shape under the Introduction section 1 of the main body of the manuscript. All suggested corrections have been implemented. Please see the revised introduction in main body of the manuscript. |
| 19. Methodology Please, replace “Methodology” with “Methods and materials.” | Thank you! We made the required changes. |
| 20. Study population I suggest omitting unnecessary qualifiers from the document, such as “carefully” in the below statement. “The study carefully outlined its target population, establishing precise criteria for inclusion and exclusion to emphasize the unique characteristics of the participants.” | Yes, it is advisable to omit unnecessary qualifiers to maintain clarity and conciseness. Please see the revised text in the main body of the manuscript under section 2.3. Study Population. |
| 21. Inclusion criteria Mention how “legal wedding” status of couples was ascertained to include/exclude participants. | Thank You! To ascertain the "legal wedding" status of couples for inclusion/exclusion, the study utilized local marriage registries and community verification processes. This ensured that only legally recognized marriages were considered, thereby maintaining the integrity of the study population. |
| 22. The criterion, “with consistent mobility for at least a year” also seems vague, unless the purpose of mobility is attached to a pastoralist life style, e.g., in search of grass and water for their cattle, etc. In other words, what if couples were consistently mobile for trading or some other purpose unrelated to a pastoralist life style? | Thank you! The criterion "with consistent mobility for at least a year" specifically refers to mobility associated with a pastoralist lifestyle, such as movements in search of grass and water for livestock. This clarification is crucial to ensure that the study population accurately reflects the unique characteristics of pastoralist communities. Couples who are consistently mobile for trading or other purposes unrelated to pastoralism would not meet this specific inclusion criterion. Please see the revised text in the main body of the manuscript under section |
| 23. Study Population. What the authors meant by “mobile regions” is not clear. | The term "mobile regions" refers to areas where pastoralist communities frequently move due to their lifestyle, primarily in search of grass and water for their livestock. These regions are characterized by nomadic or semi-nomadic patterns of movement, which are essential for the survival of their livestock. This clarification ensures that the study population accurately reflects the unique characteristics of pastoralist communities. On the other hand, the Pastoralist community is not only mobile within the Study area (Fentale District). For example, they move to the Afar region in search of pasture and water for their livestock. This means that the research team was moving together in the direction the pastoralists were moving during the research period. This approach allowed the study to capture the dynamic nature of the pastoralist lifestyle and ensure that the findings accurately reflected their unique challenges and needs. |
| 24. The criterion, “couples committed to remaining in the district or mobile areas for at least a year and a half from the data collection period” similarly lacks clarity. More specifically, the criterion “couples committed to remaining in the district or mobile areas” seems unjustifiable as this was a cross sectional, and not a longitudinal study. The phrase “mobile areas” is unclear here as well. | Although this study is cross-sectional, it complements or is interdependent with a longitudinal or interventional study design. This study serves as a baseline for future intervention studies. Additionally, "mobile areas" refers to "mobile regions." We have defined the term "mobile regions" correctly earlier in the document. |
| 25. The paper should provide a strong argument for using “husbands consenting for their wives' participation” as a criterion in light of the global standards for ethical conduct of researches involving human subjects. For example, husband’s consent is mandatory for certain studies involving greater than minimal risk to subjects, especially pregnant women and/or babies. | In research involving human subjects, especially in contexts with cultural and ethical complexities, it's essential to balance local norms with global ethical standards. In this study, verbal informed consent was obtained from spouses acting on behalf of wives under 18, aligning with cultural norms and ethical guidelines. This approach recognizes the cultural context where husbands' consent is considered appropriate, particularly in studies involving minimal risk to subjects. The paper should provide a strong argument for this practice by highlighting that husband’s consent is often mandatory for studies involving greater than minimal risk, especially those concerning pregnant women and/or babies. This justification ensures that the research adheres to both local cultural practices and the global standards for ethical conduct in research involving human subjects. Please refer to the revised text in the main body of the manuscript under section 2.8, Ethical Considerations. |
| 26. The exclusion criteria should similarly be revisited in light of the comments for the inclusion criteria. | Thank you for your comment. We have revisited the exclusion criteria to ensure they align with the revised inclusion criteria. This includes clarifying any ambiguous terms and ensuring that the criteria are justified based on the study design and ethical considerations. |
| 27. Sample size determination Similar to the previous comment, I suggest omitting the phrase “meticulous calculation” as it is an unnecessary qualifier. | Thank you! We have made the required changes. |
| 28. For logical reasons and to avoid redundancies, I suggest merging this section with the subsequent section.  Study design, time frame, and sampling approach For logical reasons, the overarching “Study design” bits of this section should be moved up next to study setting/area. Because design is an umbrella theme encompassing population, participant recruitment criteria, sampling design, data collection, analysis, …. | Thank you! We have made the required changes. |
| 29. Describing “Study population” earlier at the very top of the section, and “source population” in this sub-section (see 5th statement) is another illustration of incoherence and fragmentation in the paper. Please, refer to the previous comment in the abstract about multi-stage sampling strategy. | Please refer to Fig 1 for the multi-stage sampling strategy, as previously mentioned in the Abstract. |
| 30.Data collection The section is provided in about half a page long description which is only 1 paragraph, which in turn, is composed of 22 lines. This is another illustration of the prevailing writing issues in the paper | I believe what is stated about this data collection is correct. There is something that should not be left out. There are 9 lines instead of 22 lines in the first paragraph. |
| 31. Data management and analysis approach Refer to the previous similar comments about the use of “meticulously”. | We have made the required changes. |
| 32.Refer to the previous comment in the abstract about the use of both SPSS and STATA. Just mentioning that they were used for comprehensive analysis doesn’t look adequate. For instance, which variables, models, or aspects of analyses required SPSS features that STATA lacks, and/or vice versa?  Refer to the previous comment in the abstract about the use of both SPSS and STATA. Just mentioning that they were used for comprehensive analysis doesn’t look adequate. For instance, which variables, models, or aspects of analyses required SPSS features that STATA lacks, and/or vice versa? | We have made the necessary changes in Section 2.7, Data Management and Analysis Approach, in the body of the manuscript. |
| 33. Multicollinearity was assessed using the VIF, confirming the integrity of the data structure with no significant multicollinearity present.” – Please, refer to the previous comment in the abstract about the potential for multicollinearity among/between a number of independent variables. | We have made the necessary changes in Section 2.7, Data Management and Analysis Approach, in the body of the manuscript. |
| 34. Measures: Pastoralism in Ethiopia For logical reasons, this section should precede Data Analysis. | We have made the required changes. |
| 35. As commented earlier in the abstract and afterwards, the paper doesn’t define the concept “nomadic pastoralism”, even in this section, despite its repeated use. Instead, this section defines “agro-pastoralism” which has never been used so far. | Thank you! The distinction between nomadic pastoralism and agro-pastoralism is already outlined in section 2.7, "Measures." Given the detailed context of pastoralism in Ethiopia, it's crucial to closely consider this distinction. |
| 36.What the authors meant by “active FP method utilizer” | **“**Active FP method utilizer" likely refers to an individual or a group of individuals who actively use a family planning (FP) method to prevent or delay pregnancy. This term denotes consistent and ongoing use of contraception or other family planning methods, suggesting a proactive approach to managing reproductive health and family size. |
| 37. “The challenges confronting this predominant way of life include high population trends, prolonged resource conflicts, and restricted access to grazing lands and drinking water. These challenges are often attributed to the impacts of climate change and the prevalence of animal diseases [45].” | This section holds significant importance as it addresses our understanding of pastoralism and its associated challenges, which are key focuses of this study. For instance, the high population profile and resource constraints underscore the importance of promoting family planning. Additionally, the volatile climate contributes to high child mortality rates, influencing couples to desire more children. |
| 38.Ethical consideration Refer to the previous comment under inclusion criteria about ethical issues peculiar to this study | We have accepted and incorporated the suggested modification accordingly. |
| 39. Results, discussion, conclusion and recommendations The authors need to revisit these sections in light of the comments provided in the previous sections as appropriate. | We have accepted and incorporated the suggested modification accordingly. |
| 40. Contradicting funding information The “Disclosure” statement reads as follows: “The funding organization played no role in the study design, data collection, analysis, interpretation, protocol writing, or submission.” This information contradicts with another statement saying, “Supporting Information confirming no funding was granted and …..” | Thank you for your comment. We acknowledge the oversight regarding the "Disclosure" statement and have now removed it from the manuscript. |
| Reviewer#2: Review Feedback | **Authors’ responses** |
| 1.General comment  Title: Bridging Disparity in Knowledge and Utilization among Married Couples in the Pastoralist Community of Fentale District, Eastern Ethiopia General comments: The findings of this study have a significant input to find out the Disparity in Knowledge and Utilization of contraceptive among Married Couples in the Pastoralist. As there are no studies on contraceptives specifically among the pastoralists, the outcomes of this study can be used to set a baseline for the intervention and to conduct furthers study. | Response: Thank you for your thorough review of our manuscript. We have made the necessary revisions, and we believe the outcomes of this study can now be used to set a baseline for interventions and further research. We appreciate your feedback and are confident these changes have strengthened our manuscript. If you have any additional suggestions, please let us know. |
| 2. The core ideas of the research should be mentioned in the title of the research. It is about what? Family planning? Contraceptive? | Yes, we have changed the title of our manuscript to "Bridging Disparity in Knowledge and Utilization of Contraceptive Methods among Married Couples in the Pastoralist Community of Fentale District, Eastern Ethiopia" to better reflect the core ideas of the research. We have included the term "Contraceptive Methods" to specify the focus of the study. |
| 3.Abstract • In the introduction part of the abstract, the gap of study and objectives should be mentioned | Thank you! We have explained the objective of this study correctly in the last paragraph of the Introduction section of the manuscript. Adding it to the introduction of the abstract would exceed the word limit set by the PLOS ONE journal. However, the study gap has already been addressed in the introduction of the abstract, and the detailed study gaps have been explained in the Introduction section of the manuscript. |
| 4.Abstract • Methods: ‘‘….Data entered into EPI Data underwent analysis with SPSS (v23.0) and STATA (v14.0), employing descriptive statistics, bivariate analysis, and binary logistic regression to identify predictors of contraceptive knowledge and utilization.’’ what models are analyzed by both SPSS and STATA? | Thank you! Here’s a detailed response that outlines the specific advantages of using both SPSS and STATA, examples of analyses suited to each software, and how their functionalities complement each other in this research. This information has been incorporated into the body of the manuscript under section 2.7, titled "Data Management and Analysis Approach," specifically in paragraphs 2 and 3. |
| 5. Abstract • ‘‘Results: This couple-based cross-sectional study, conducted between October 1 and December 25, 2021, explores knowledge and utilization variations among 1496 couples in the pastoralist community.’’ Don’t repeat the same points in the abstract. For example: …conducted between October 1 and December 25, 2021…. among 1496 couples in the pastoralist community. | Thank you for the suggestion. Your input is essential. We have corrected the duplication by deleting it from the results section in the abstract. Additionally, we have revised the structure of the abstract's results section to ensure clarity and conciseness. |
| 6.Abstract  Results  • Incomplete idea… exhibits educational disparities (53.8% with no formal education). What is that disparity? | Thank you! We agree with the comment and have edited the text accordingly (please see the results section of the abstract). |
| 7.Abstract  Results  • Is that not only women who utilize contraceptive …. ‘‘Women (41.2%) surpass men in contraceptive utilization.’’ | Thank you! We agree with the comment and have edited the text accordingly (please see the results section of the abstract). |
| 8. What percent of participants get information from HEW? ‘‘…Health extension workers play a significant role as information sources, and 44.4% of couples demonstrate comprehensive family planning knowledge’’ | Thank you! We agree with the comment and have edited the text accordingly (please see the results section 3 of the main body of the manuscript at the end of the paragraph). Because we have already revised the structure of the abstract's results section to ensure clarity and conciseness. |
| 9. Better if the author say ‘‘associations’’ rather than ‘‘correlation’’ as binary logistic reg is used. There is no negative odd ratio. ‘‘Binary logistic regression analysis reveals positive correlations between education, bank account ownership, occupation, and distance from healthcare, and media exposure with knowledge of contraception. Conversely, the nomadic-pastoralist lifestyle and specific treatment preferences exhibit negative correlations.’’ | Thank you for the suggestion. We have revised the text to use "associations" instead of "correlations," as binary logistic regression was used. We also corrected the phrasing to accurately reflect the odds ratios: "Binary logistic regression analysis reveals positive associations between education, bank account ownership, occupation, distance from healthcare, and media exposure with knowledge of contraception. Conversely, the nomadic-pastoralist lifestyle and specific treatment preferences exhibit negative associations." (Please see the results section of the abstract). |
| 10. Better if you say information rather than knowledge. ‘‘media exposure with knowledge of contraception’’ | "Media exposure with knowledge of contraception" is better. "Knowledge" implies a deeper understanding and awareness of contraception methods, which is more comprehensive than just "information." Additionally, knowledge of contraception among couples is the outcome variable of this study. |
| 11. There is a need of additional data about Disparity in Knowledge and Utilization about contraceptive, barriers, … | Yes, we have already added or corrected data in the results section of the abstract. We have revised the structure of the abstract's results section to ensure clarity and conciseness, including additional information on disparities in knowledge and utilization of contraceptives, as well as barriers to family planning. |
| 12. Introduction  First paragraph, Keep flows of idea: ‘’…… underscore the difficulties in providing services to such communities. Insights from nomadic communities in Chad [3] were crucial in addressing information gaps for tailored health services.’’ Used to address what kinds of gap? | Thank you! We have incorporated the necessary changes as suggested. |
| 13. Introduction  The second paragraph: Better if the author put at the end of introduction to show what gap the author going to address. | Thank you! We have incorporated the necessary changes as suggested. |
| 14.Introduction  Don’t repeatedly use the word …Our Study… | Thank you! We made the required changes. |
| 14.Shorten and refine the last paragraph of introduction | Thank you for your feedback. We have carefully revised the last paragraph of the introduction to ensure clarity and conciseness. |
| 16.Methodology  Study area: The third paragraph of methods seems introduction, better if is paraphrased | We have revised it to enhance clarity and coherence in presenting the study area details. |
| 17.Study Population: clearly mention the source and study population precisely | Thank you! We agree with the comment and have edited the text accordingly. |
| 18. Measures. Pastoralism in Ethiopia: Is it operational definitions? | Yes, the section titled "Pastoralism in Ethiopia" under "Measures" provides operational definitions related to pastoralism in the Ethiopian context. It delineates between nomadic-pastoralism and agro-pastoralism based on their primary economic activities—livestock herding and agriculture, respectively—and their corresponding lifestyles and resource management practices. This distinction helps frame the study's context within the specific socio-economic and environmental challenges faced by pastoralist communities in Ethiopia, including issues related to climate change, resource conflicts, and access to essential services. This version clarifies that the section indeed serves as operational definitions relevant to understanding pastoralism in Ethiopia within the study's framework. |
| 19. Knowledge of FP: Why mean is used as a reference? | The mean is used as a reference for knowledge of family planning (FP) because it provides a central, average value that summarizes the dataset, aiding in understanding the typical level of knowledge across participants. This approach assumes the data is normally distributed or not skewed either to the left or right. |
| 20. Ethical Consideration: The right place? Check the standard format of PLOS ONE | Yes, I am certain that the Ethical Considerations are explained in the appropriate section. |
| 21. Results  Check grammar and flow of idea. Don’t repeat the same words repeatedly. | Thank you for your comments. We have revised and corrected the grammar and flow of ideas in the results section of the manuscript |
| 22. ‘‘Results: Marital Couples: Socio-demographic and Reproductive Disparities:’’ Make it precise | Thank you! We made the required changes. |
| 23.Results  Focus on your outcome variable, briefly explain your finding, and don’t make it lengthy. | Thank you for your suggestion. We have focused on the outcome variable and provided a brief explanation of our findings. |
| 24.Results  Generally, the result part needs intensive revision; the author should focus on the objective of the study. Check the grammar and shorten the lengthy paragraphs. Discussion: shallow and highly fragmented. | Thank you for your suggestion. We have revised the results section intensively, focusing on the study's objectives. We have also provided a concise explanation of our findings. |

Manuscript is revised and attached.
